# Role of the dorsal attention network in distracter suppression based on features

**DOI:** 10.1080/17588928.2019.1683525

**Published:** 2019-11-01

**Authors:** Armien Lanssens, Gloria Pizzamiglio, Dante Mantini, Celine R. Gillebert

**Affiliations:** a Department of Brain and Cognition, KU Leuven, Leuven, Belgium; b Department of Experimental Psychology, University of Oxford, Oxford, UK; c Research Center for Motor Control and Neuroplasticity, KU Leuven, Leuven, Belgium; d Brain Imaging and Neural Dynamics Research Group, IRCCS San Camillo Hospital, Venice, Italy

**Keywords:** fMRI, attentional priority, feature-based attention, dorsal attention network, frontal eye fields, intraparietal sulcus, superior parietal lobe

## Abstract

Selective attention allows us to prioritize the processing of relevant information and filter out irrelevant information. Human functional neuroimaging and lesion-based studies have highlighted the fronto-parietal dorsal attention network (DAN) as an important network in this process. In this study, we investigated the role of the DAN in distracter suppression by dynamically modifying the priority of visual information (target > high priority distracter > low priority distracter) based on features only. To this end, we collected fMRI data in 24 healthy subjects, who performed a feature-based variant of the sustained attention to response task. Participants had to select one or attend two stream(s) of overlapping digits that differed in color and respond to each digit in the task-relevant stream(s) except to a single non-target digit. Results showed higher DAN activity when a target was co-presented with a high versus low priority distracter. Furthermore, higher DAN activity was observed when selectively attending one (target + high/low priority distracter) versus simultaneously attending two (target + target) stream(s) of digits. In conclusion, our study highlights the contribution of the DAN in the feature-based suppression of task-irrelevant information.

## 1. Introduction

Selective attention allows us to prioritize the processing of behaviorally relevant information and to filter out irrelevant information (Desimone & Duncan, 1995). To this end, the attentional priority of perceptual units in our visual field is continuously adapted (Bundesen, 1990; Ptak, 2012; Vandenberghe, 2015) based on bottom-up (physical salience) as well as top-down (behavioral relevance) factors (Koch & Ullman, 1985; Theeuwes, 2010). The top-down allocation of attention can be based on spatial locations (e.g. the left side of a room) (Posner, 1980), objects (e.g. a balloon) (Duncan, 1984), or even on specific features of objects (e.g. the color red) (Driver & Baylis, 1989; Egeth & Yantis, 1997; Maunsell & Treue, 2006). Given the limited capacity of our information processing resources (Broadbent, 1957), stimuli with a high priority will be preferentially processed at the cost of stimuli with a low priority (Itti & Koch, 2001; Jerde & Curtis, 2013).

Multiple brain regions have been reported to play a role in selective attention. Several of these regions cluster into the fronto-parietal dorsal attention network (DAN). Besides a motion-sensitive region in the middle temporal cortex (MT+/V5), the DAN consists of regions in the frontal (frontal eye field) and posterior parietal (intraparietal sulcus, superior parietal lobe) cortex, each of which contributes to the process of selective attention (Corbetta & Shulman, 2002; Manohar, Bonnelle, & Husain, 2014).

The frontal eye fields (FEF) have been argued to contain a representation of the priority of items in the visual field to plan and execute eye movements (Bichot & Schall, 1999; Bruce & Goldberg, 1985; Jerde & Curtis, 2013; Neggers et al., 2007; Schall & Thompson, 1999; Thompson, Bichot, & Schall, 1997), and may therefore play a critical role in filtering out irrelevant visual information (Hung, Driver, & Walsh, 2011; Lega et al., 2019). The main function of the superior parietal lobe (SPL) is shifting the focus of attention (Molenberghs, Mesulam, Peeters, & Vandenberghe, 2007; Vandenberghe, Molenberghs, & Gillebert, 2012) between spatial locations (Corbetta, Kincade, Lewis, Snyder, & Sapir, 2005; Vandenberghe & Gillebert, 2009; Vandenberghe, Gitelman, Parrish, & Mesulam, 2001; Yantis et al., 2002) and features of objects (Greenberg, Esterman, Wilson, Serences, & Yantis, 2010; Liu, Slotnick, Serences, & Yantis, 2003). On the other hand, the intraparietal sulcus (IPS) seems to play a key role in the selection among competing stimuli (Bundesen, Habekost, & Kyllingsbæk, 2005; Molenberghs, Gillebert, Peeters, & Vandenberghe, 2008; Silver & Kastner, 2009; Vandenberghe et al., 2005). Neuroimaging studies in neurologically healthy participants revealed that the simultaneous presentation of a target and a distracter results in higher IPS activity than that of one isolated target (Gillebert et al., 2012a; Gillebert, Mantini, Peeters, Dupont, & Vandenberghe, 2013; Molenberghs et al., 2008). Similar findings were obtained in studies where either the number (Gillebert et al., 2012b; Guo, Preston, Das, Giesbrecht, & Eckstein, 2012; Maximo, Neupane, Saxena, Joseph, & Kana, 2016; Nobre, Coull, Walsh, & Frith, 2003) or the priority (Anderson et al., 2007; Bardi, Kanai, Mapelli, & Walsh, 2013; Mevorach, Hodsoll, Allen, Shalev, & Humphreys, 2010; Mevorach, Humphreys, & Shalev, 2006, 2009; Sapountzis, Paneri, & Gregoriou, 2018; Sui, Liu, Mevorach, & Humphreys, 2015; Wei, Yu, Müller, Pollmann, & Zhou, 2018) of distracters was increased. In the latter case, a distracter can be of high priority in a purely bottom-up fashion (e.g. increased physical salience) or through top-down processes (e.g. increased number of shared features with a goal-relevant item) (Ptak, 2012). Increasing either the number or the priority of distracters challenges the process of selective attention, since it needs to be precise to successfully resolve the competition for processing resources between task-relevant and task-irrelevant visual information (Itti & Koch, 2001).

Although the DAN is involved in each perceptual dimension of selective attention, suppressing distracters based on features seems to rely on partially different neural mechanisms than, and to operate independently from, suppressing distracters based on spatial locations (Hou & Liu, 2012; Liu & Hou, 2013; Wildegger, van Ede, Woolrich, Gillebert, & Nobre, 2017). Here, the DAN appears to be differentially activated when selecting visual information based on either spatial locations or features (Giesbrecht, Woldorff, Song, & Mangun, 2003; Greenberg et al., 2010). Furthermore, selection based on features appears to originate in the prefrontal cortex (Bichot, Heard, DeGennaro, & Desimone, 2015) and selecting different kinds of features can activate distinct neuronal subpopulations in the visual cortex (Liu, Hospadaruk, Zhu, & Gardner, 2011; Zanto, Rubens, Bollinger, & Gazzaley, 2010). Thus, as the majority of research on selective attention contains spatial components (e.g. Bundesen et al., 2005; Gillebert et al., 2012a, 2012b, 2013; Maximo et al., 2016; Molenberghs et al., 2008, 2007; Nobre et al., 2003; Sapountzis et al., 2018; Vandenberghe et al., 2005, 2012; Wei et al., 2018), studies incorporating only the perceptual dimension of features are warranted.

In the current study, we aimed to investigate the role of the DAN in suppressing distracters by dynamically modifying the attentional priority of visual information based on features only. To this end, participants performed a feature-based variant (Finsterwalder, Demeyere, & Gillebert, 2017) of the sustained attention to response task (SART) (Robertson, Manly, Andrade, Baddeley, & Yiend, 1997). This paradigm has an innovative design, in which the priority of two stimuli dynamically changes over time while the physical salience is kept constant (Molenberghs et al., 2007; Vossel et al., 2014). Furthermore, as a continuous performance task, the feature-based SART provides a sustained attention baseline for the conditions of interest, i.e. to eliminate effects related to deficits in sustained attention (e.g. Liu et al., 2003; Molenberghs et al., 2007). We hypothesized that, when selectively attending one task-relevant stream of visual information, DAN activity would be increased by raising the priority of the distracter in the task-irrelevant stream. Further, we hypothesized that DAN activity would be increased by selectively attending one task-relevant (while suppressing one task-irrelevant) stream of visual information as opposed to simultaneously attending two task-relevant stream(s) of visual information. These hypotheses are based on results of aforementioned studies showing increased DAN activity when challenging selective attention, however, not (only) relying on the perceptual dimension of features.

## 2. Methods

### 2.1. Participants

24 healthy young participants (13 female, 20–36 years, all right-handed) were recruited through the Oxford Psychology recruitment scheme. All participants had normal or corrected-to-normal vision and did not suffer from color blindness. The study was approved by the Medical Science Interdivisional Research Ethics Committee, University of Oxford and all participants gave written informed consent in accordance with the Declaration of Helsinki.

### 2.2. Task and stimuli

**Figure 1.**
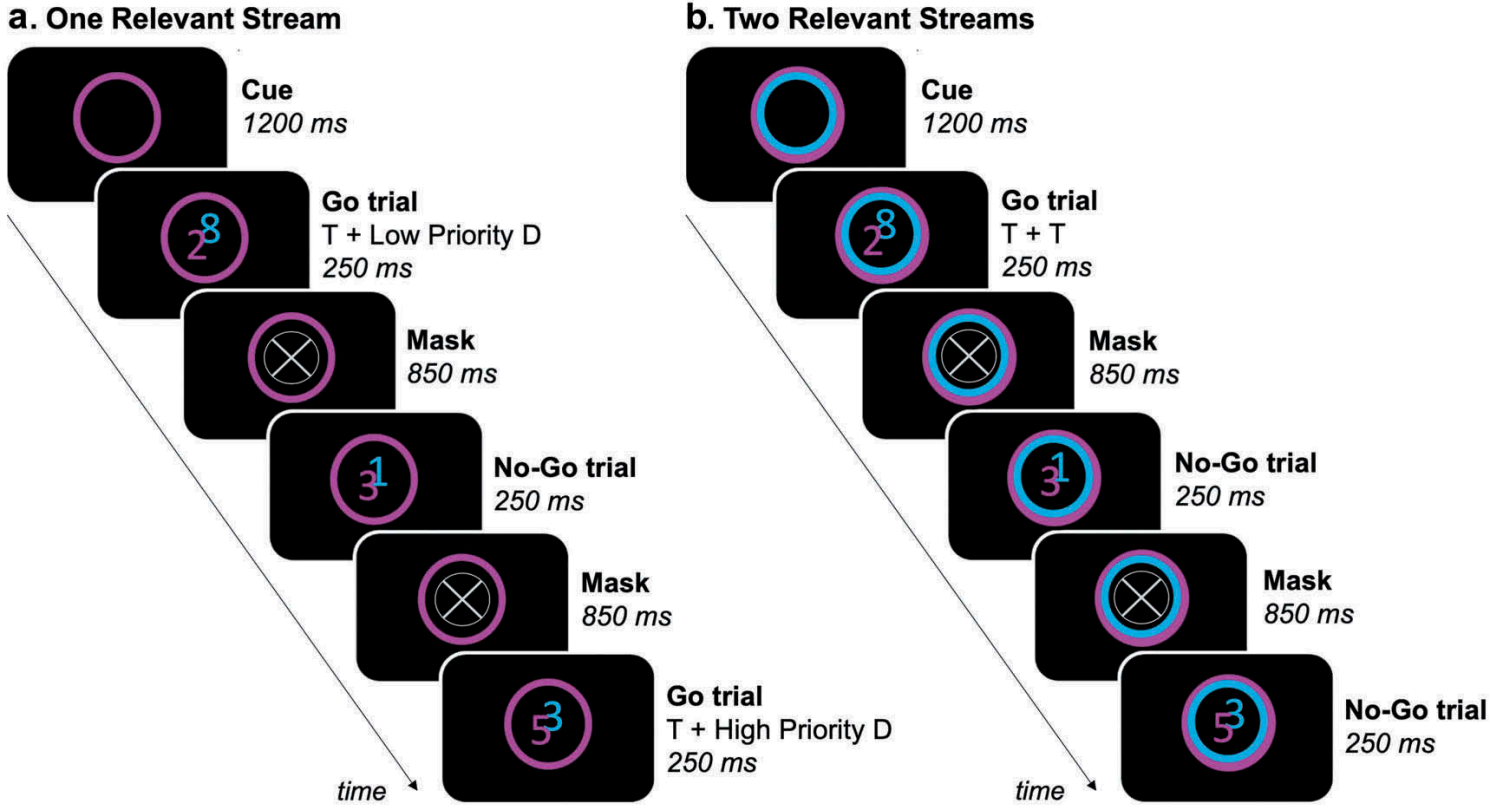
Feature-based variant of the sustained attention to response task. The task-relevant stream(s) was/were indicated by a colored circle. Participants were instructed to press a button on a response box when a target (digit ≠ ‘3ʹ) appeared in the task-relevant stream(s) (= go trials), but had to inhibit the response when a non-target (digit = ‘3ʹ) appeared in a task-relevant stream (= no-go trials). (a) In experimental blocks where only one stream of digits was task-relevant, two types of go trials occurred: target (T) (task-relevant digit ≠ ‘3ʹ) with high (task-irrelevant digit = ‘3ʹ) or low (task-irrelevant digit ≠ ‘3ʹ) priority distracter (D). (b) In experimental blocks where both streams of digits were task-relevant, only one type of go trial occurred (T + T) (both digits ≠ ‘3ʹ).

Stimulus presentation and response registration were controlled by a personal computer running Presentation 17.0 (Neurobehavioural Systems). Stimuli were projected onto a screen (ST-Professional-DC, Screen-Tech, using a CHRIS-TIE Mirage S + 2K projector (100 Hz, 1400 × 1050 pixel resolution). Responses were given using a custom-made MRI-compatible response box. A feature-based variant (Finsterwalder et al., 2017) of the SART (Robertson et al., 1997) was performed during fMRI data acquisition (Figure 1). In each task trial, two partly overlapping equiluminant streams of digits were displayed in the center of a black screen for 250 ms. One stream was colored in cyan, the other one in magenta (digit size = ~0.80°). A cue-only trial occurred before each experimental block, where a cue was presented as a colored circle (diameter = 1.60°) for 1200 ms. This cue remained on the screen during the presentation of digits and indicated the task-relevant stream(s). Three different cues were possible: a single cyan or magenta colored circle indicating which of the two streams was task-relevant, or a both cyan and magenta colored circle indicating that both streams were task-relevant. The digits ranged from one to nine and were presented sequentially in random order. A mask followed each presentation of digits for 850 ms. The mask consisted of a ring (diameter = ~1.60°) with a diagonal cross in the middle.

We used a mixed experimental design, with each participant completing two fMRI runs each consisting of 18 experimental blocks. There were six experimental blocks where the magenta stream was task-relevant (and the cyan stream had to be suppressed), six where the cyan stream was task-relevant (and the magenta stream had to be suppressed) and six where both streams were task-relevant. Each sequence of three experimental blocks contained each of these situations exactly once in a fixed order within runs. After each sequence of three experimental blocks, a rest period of 12 s occurred. The order of the experimental blocks was counterbalanced across runs and participants.

Each experimental block consisted of 18 task trials, resulting in a total of 324 task trials per run. To avoid a (micro-)spatial bias while performing the task, the magenta and cyan stream randomly switched sides after each three consequent trials. This resulted in half of the trials within each experimental block where the magenta stream was on the left and the cyan stream on the right, and the other way around for the other half of the trials. Participants were instructed to press a button on the response box every time a target (digit ≠ ‘3ʹ) appeared in the task-relevant stream(s) (= go trials). However, participants had to inhibit the response when a non-target (digit = ‘3ʹ) appeared in a task-relevant stream (= no-go trials) (Table 1). In each sequence of nine trials, each stream contained each possible digit exactly once in random order, with the restriction that co-presented digits were never the same. This implies that each experimental block contained two no-go trials (task-relevant digit = ‘3ʹ), while all other task trials were go trials (task-relevant digit(s) ≠ ‘3ʹ). For each experimental block where only one stream of digits was task-relevant, this also implies that two go trials occurred where the digit ‘3ʹ was present in the task-irrelevant stream. The digit ‘3ʹ in the task-irrelevant stream was considered a high priority distracter, since it had the same identity (but a different color) as a non-target in the task-relevant stream, in contrast to the other digits in the task-irrelevant stream (low priority distracters).

**Table 1.** Experimental conditions.

Experimental block	Experimental condition	Number of trials per run	Relevant stream(s)	Irrelevant stream	Trial type
One Relevant Stream	T + Low Priority D	168 (7/9)	1 Target (≠ 3)	1 Low Priority Distracter (≠ 3)	Go
	T + High Priority D	24 (1/9)	1 Target (≠ 3)	1 High Priority Distracter (= 3)	Go
	Non-Target	24 (1/9)	1 Non-Target (= 3)	1 Low Priority Distracter (≠ 3)	No-Go
Two Relevant Streams	T + T	96 (8/9)	2 Targets (≠ 3)	N.A.	Go
	Non-Target	12 (1/9)	1 Non-Target (= 3) & 1 Target (≠ 3)	N.A.	No-Go

For experimental blocks where only one stream of digits was task-relevant, three experimental conditions occurred: Target (T) + Low Priority Distracter (D), Target (T) + High Priority Distracter (D), and Non-Target. For experimental blocks where both streams of digits were task-relevant, two experimental conditions occurred: Target (T) + Target (T), and Non-Target.

Participants were asked to fixate the center of the screen, avoiding eye movements, and to pay attention to both speed and accuracy while performing the task. In this study, fixation control was not possible since eye-tracking data could not be obtained under fMRI conditions. However, the occurrence of spatial-based attentional processes is unlikely considering that stimuli were presented at the fovea and subjects were instructed to avoid eye movements.

### 2.3. Image acquisition

Structural and functional magnetic resonance images were acquired through a 3T Siemens TIM Trio scanner equipped with a 32-channel head coil array. A high-resolution structural scan was obtained through a T_1_-weighted three-dimensional turbo-field-echo sequence (repetition time = 2040 ms, echo time = 4.70 ms, flip angle = 8 degrees, 1.00 mm isotropic resolution). 152 whole-brain functional MRI volumes were continuously acquired using a single-shot echo-planar imaging sequence (repetition time = 3000 ms, echo time = 30 ms, flip angle = 87 degrees, 3.00 mm isotropic resolution).

### 2.4. Data analysis

fMRI data processing was performed using Statistical Parametric Mapping (SPM12) software (http://www.fil.ion.ucl.ac.uk/spm/) and custom-made scripts written in MATLAB (MATLAB 9.0, The MathWorks Inc., Natick, MA). Behavioral data analysis was performed using R (R 3.5.3, R Development Core Team, 2016) and the lme4 package (lme4 1.1.21) (Bates, Mächler, Bolker, & Walker, 2015). Data analyses were based on 19 out of 24 recruited participants. One participant was excluded based on noncompliance with the task (> 5% misses on all go trials and > 20% outliers in reaction times). Additionally, one participant was excluded based on excessive head motion (> 4 mm translation and > 5° rotation) (Johnstone et al., 2006), and three participants were excluded due to technical issues in the acquisition/reconstruction of fMRI data.

#### 2.4.1. Behavioral data analysis

To determine the behavioral effect of dynamically modifying the priority of visual information, both accuracy and reaction times (RTs) were compared between the different experimental conditions of go trials.^1^ 1Data on no-go trials were not discussed in detail. The proportion of false alarms (no-go trials where participants erroneously pressed the button) was .34 when one stream had to be attended selectively and .25 when both streams had to be attended simultaneously. Here, accuracy levels were analyzed using a Poisson mixed-effects model. The accuracy is reflected through the proportion of misses, where a miss is defined as a go trial where participants erroneously did not press the button. On the other hand, RTs were analyzed by using a linear mixed-effects model. At the individual level, for each experimental condition, trials with RTs exceeding M ± 3 x SD were defined as outliers and excluded from the behavioral analysis.

#### 2.4.2. fMRI data analysis

For each included participant, the first two functional images of both runs were excluded from preprocessing and subsequent statistical analysis (Diedrichsen & Shadmehr, 2005). The preprocessing of fMRI data was performed by realigning the individual functional images with the mean image of each run to correct for head movements, followed by co-registration with the T_1_-weighted image. The T_1_-weighted image was warped into Montreal Neurological Institute (MNI) space (via the segmentation option in SPM) and the resulting transformation was used to spatially normalize the functional MR images. The images were spatially smoothed with an 8 mm full-width half-maximum kernel. For each run, the general linear model (GLM) contained all five experimental conditions and six motion regressors (one motion parameter for either translation or rotation in each of the three dimensions of space). Importantly, cue-only trials and incorrect responses to trials were modeled separately. This implies that further mentioning of the five different experimental conditions in the context of fMRI only indicates the correct responses to the respective trials. At the modeling stage, a time derivative was added to capture the delay in the hemodynamic response. Next, the parameters were estimated for each voxel, resulting in the GLM of the blood-oxygen-level dependent signal for each participant individually.

To answer our research question, contrasts between experimental conditions were calculated for equal voxels of the functional MR images.^2^ 2In the current study, brain activity elicited by no-go trials was not investigated since it would be confounded by their rare and unexpected appearance, and by response inhibition. Here, a paired *t*-test was used to determine whether activity in a respective voxel was stronger in one of both experimental conditions. To investigate the effect of increasing distracter priority, trials where a target was co-presented with a high priority distracter were contrasted with those where a target was co-presented with a low priority distracter (Contrast 1). On the other hand, to investigate the effect of selecting one relative to attending two stream(s) of stimuli, trials where a target was co-presented with a low priority distracter were contrasted to those where two targets were co-presented (Contrast 2) and trials where a target was co-presented with a high priority distracter were contrasted to those where two targets were co-presented (Contrast 3).

In a group-level statistical analysis, a random-effects model was estimated. Results were presented as statistical parametric maps relying on cluster-extent based thresholding. A primary voxel-level threshold was set to uncorrected *p* < .001 and a secondary cluster-level threshold was set to *p* < .05, corrected for multiple testing using the family wise error (FWE) method (Poline, Worsley, Evans, & Friston, 1997).

## 3. Results

### 3.1. Behavioral results

#### 3.1.1. Accuracy

**Figure 2.**
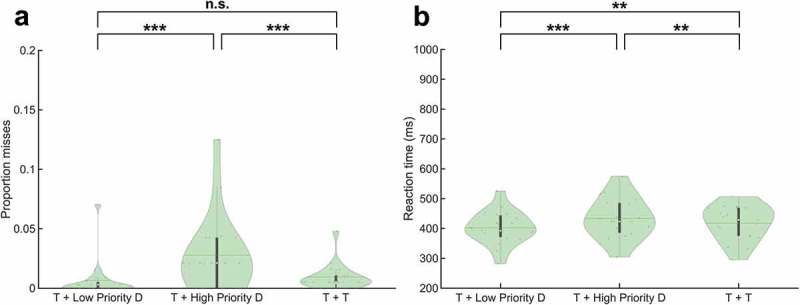
Behavioral results. (a) Proportion misses, and (b) Reaction times on go trials. * = *p* < .05, ** = *p* < .01, *** = *p* < .001, n.s. = not significant (*p* > .05). Abbreviations: ‘T’ = Target, ‘D’ = Distracter.

Behavioral data showed significantly more misses when a target was co-presented with a high priority distracter relative to a low priority distracter (*Z* = 4.81, *p* = 1.49 x 10^−6^) and when a target was co-presented with a high priority distracter relative to co-presenting two targets (*Z* = 4.55, *p* = 5.45 x 10^−6^) (Figure 2(a)). No significant difference in the proportion of misses was found between the co-presentation of a target and a low priority distracter and the co-presentation of two targets (*Z* = 0.69, *p* = .49).

#### 3.1.2. Reaction times

RTs were significantly increased when responding to a target co-presented with a high priority distracter relative to a low priority distracter (*t*(36)* = *5.53, *p* = 2.93 x 10^−6^), when responding to a target co-presented with a high priority distracter relative to responding to two co-presented targets (*t*(36)* = *2.79, *p* = 8.45 x 10^−3^) and when responding to two co-presented targets compared to responding to a target co-presented with a low priority distracter (*t*(36)* = *2.75, *p* = 9.35 x 10^−3^) (Figure 2(b)).

### 3.2. fMRI results

#### 3.2.1. Contrast 1: suppressing a high versus low priority distracter

**Figure 3.**
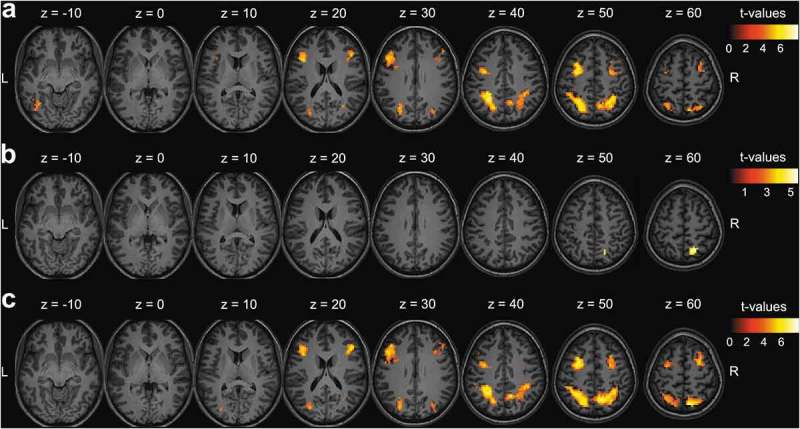
fMRI results. (a) Contrast 1: effect of increasing distracter priority, (b) Contrast 2: effect of selecting one (low priority distracter) versus attending two stream(s) of stimuli, and (c) Contrast 3: effect of selecting one (high priority distracter) versus attending two stream(s) of stimuli. Results of correct responses on go trials presented as statistical *t*-maps in MNI (Montreal Neurological Institute) space. Cluster-extent based thresholding was used with voxel-based threshold uncorrected *p* < .001 and cluster-based threshold FWE (familywise error)-corrected *p* < .05.

When selectively attending one stream of digits, activity in the fronto-parietal DAN was dynamically modulated by the identity of the distracter in the task-irrelevant stream. More specifically, co-presenting a target with a high priority distracter relative to a low priority distracter resulted in significantly higher activity for bilateral IPS and SPL, bilateral FEF, bilateral middle frontal gyri and left MT+ (Figure 3(a) and Table 2A).

**Table 2.** Significant clusters in (a) Contrast 1: effect of increasing distracter priority, (b) Contrast 2: effect of selecting one (low priority distracter) versus attending two stream(s) of stimuli, and (c) Contrast 3: effect of selecting one (high priority distracter) versus attendingtwo stream(s) of stimuli.

Contrast	Region	x, y, z (MNI)	Number of voxels (27 mm^3^)	Peak *Z*-score	FWE-corr. *p*
a	Left IPS + SPL	−27, −52, 44	546	5.01	<.001
	Left FEF + MFG	−27, 2, 50	404	4.70	<.001
	Right IPS + SPL	12, −58, 38	465	4.53	<.001
	Right FEF	30, 8, 56	112	4.09	<.001
	Right MFG	48, 29, 26	70	3.91	.002
	Left MT+	−39, −61, −13	39	3.73	.032
b	Right SPL	15, −61, 59	69	4.18	.004
c	Left IPS + SPL	−27, −52, 44	687	5.08	<.001
	Left FEF + MFG	−27, 2, 50	522	4.89	<.001
	Right IPS + SPL	9, −70, 59	646	4.88	<.001
	Right FEF	30, 8, 56	183	4.59	<.001
	Right MFG	48, 29, 23	117	4.52	<.001

For each significantly activated cluster, the anatomical region, stereotactic MNI (Montreal Neurological Institute) coordinates, number of voxels, peak *Z*-score and FWE (familywise error)-corrected *p*-value is reported. Here, the FWE-corrected *p*-value was calculated based on the number of voxels in each cluster. Cluster-extent based thresholding was used with primary threshold uncorrected *p* < .001 and FWE-corrected *p* < .05. Abbreviations: IPS = intraparietal sulcus, SPL = superior parietal lobe, FEF = frontal eye fields, MFG = middle frontal gyrus, MT+ = middle temporal.

#### 3.2.2. Contrast 2 & 3: selecting one versus attending two stream(s) of stimuli

Selectively attending one stream as opposed to simultaneously attending two streams of digits modulated activity in the fronto-parietal DAN. More specifically, right SPL activity was significantly higher when selecting a target co-presented with a low priority distracter than when simultaneously attending two targets (Figure 3(b) and Table 2B). Furthermore, activity in the bilateral IPS and SPL, bilateral FEF and bilateral middle frontal gyri was significantly higher when selecting a target co-presented with a high priority distracter than when simultaneously attending two targets (Figure 3(c) and Table 2C).

## 4. Discussion

In this study, we investigated the role of the DAN in distracter suppression by dynamically modifying the priority of visual information based on features only. Specifically, we examined whether DAN activity was modulated by the attentional priority of the distracter, and whether DAN activity was modulated by the need to either select one or attend two stream(s) of stimuli. Results showed higher DAN activity when a target was co-presented with a high priority distracter relative to a low priority distracter (Figure 3(a) and Table 2A). Further, higher activity in a subregion of the DAN (right SPL) was observed when selectively attending a target co-presented with a low priority distracter compared to simultaneously attending two targets (Figure 3(b) and Table 2B). Finally, higher DAN activity was observed when selectively attending a target co-presented with a high priority distracter compared to simultaneously attending two targets (Figure 3(c) and Table 2C).

### 4.1. Suppressing a high versus low priority distracter

Despite being instructed to selectively attend one stream of digits and suppress the other stream, performance was modulated by the features of the task-irrelevant stream. A (high priority) distracter sharing feature identity with a non-target in the task-relevant stream impaired performance compared to a (low priority) distracter with a different identity (Figure 2). The lower accuracy and increased RTs to the target are compatible with the distracter competing for information processing resources and access to visual short-term memory (VSTM) (Bundesen, 1990; McNab & Klingberg, 2008), as well as with the inhibition process triggered by the need to suppress responses to (the identity of) the distracter (Finsterwalder et al., 2017).

At the neural level, the presence of a high priority distracter relative to a low priority distracter increased the activity of the DAN. This is congruent with results of previous studies, where increasing either the number (Gillebert et al., 2012a, 2012b, 2013; Guo et al., 2012; Maximo et al., 2016; Molenberghs et al., 2008; Nobre et al., 2003) or the priority (Anderson et al., 2007; Bardi et al., 2013; Mevorach et al., 2010, 2006, 2009; Sapountzis et al., 2018; Sui et al., 2015; Wei et al., 2018) of distracters alongside one or multiple targets led to increased activity in the DAN. Furthermore, lesion-based evidence corroborates these results, where patients with parietal lesions display deficits in discriminating a target from a distracter (Friedman-Hill, Robertson, Desimone, & Ungerleider, 2003; Friedman-Hill & Wolfe, 1995; Gillebert et al., 2011). Brain stimulation of both the FEF (Hung et al., 2011; Lega et al., 2019) and IPS (Jigo, Gong, & Liu, 2018; Moos, Vossel, Weidner, Sparing, & Fink, 2012) also modified the ability to filter out distracters with varying target and distracter set sizes.

Taken together, these observations suggest that the activity of the DAN can be dynamically modulated to suppress distracters of varying priority, even when behavioral relevance is only determined by color and identity.

### 4.2. Selecting one versus attending two stream(s) of stimuli

Behaviorally, RTs were significantly higher when simultaneously attending two targets as opposed to attending a target in the presence of a low priority distracter, however, without a significant effect on accuracy (Figure 2). This finding is in agreement with previous studies, illustrating the difficulty to divide attention between multiple stimuli at the same time (Godefroy & Rousseaux, 1996; Herath, Klingberg, Young, Amunts, & Roland, 2001; Pashler, 1994). However, when the selection process was challenged by increasing the priority of the distracter, accuracy decreased and RTs increased (Figure 2).

When participants were required to selectively attend a target in the presence of a low priority distracter compared to simultaneously attending two targets, a subregion of the DAN (right SPL) displayed higher activity. Observed SPL activity can most likely be explained by its involvement in shifting attention (Corbetta et al., 2005; Greenberg et al., 2010; Liu et al., 2003; Molenberghs et al., 2007; Vandenberghe & Gillebert, 2009; Vandenberghe et al., 2001, 2012; Yantis et al., 2002), since the task-relevant and task-irrelevant stream of digits regularly switched sides. On the other hand, higher DAN activity was observed when participants were required to selectively attend a target in the presence of a high priority distracter compared to simultaneously attending two targets. This observation illustrates the involvement of the DAN in suppressing distracters based on features, which only seems to become apparent when the selection process is sufficiently challenged. Furthermore, this suggests that, in a dynamic environment where priority levels are continuously adapted, activity levels of the DAN are primarily driven by selective attention, which makes an alternative explanation of our results in terms of distracters gaining access to VSTM less likely (Linden et al., 2003; Todd & Marois, 2004, 2005; Xu, 2010; Xu & Chun, 2006). This finding is congruent with previous studies reporting a correlation between activity levels of regions in the DAN and the number of task-relevant items – but not the number of task-irrelevant items – that gain access to VSTM within each participant (Gillebert et al., 2012b; Todd & Marois, 2004; Xu & Chun, 2006), as well as with VSTM capacity across participants (Gillebert et al., 2012b; Todd & Marois, 2005). However, the design of the current study was not well suited to measure the relationship between brain activity and VSTM load. Therefore, we cannot entirely eliminate the contribution of VSTM. Furthermore, consistent with the view that attention and VSTM interact at various stages of visual processing (Gazzaley, 2011; Gillebert et al., 2012b; Lepsien, Thornton, & Nobre, 2011), several studies propose that the DAN is central to multiple (cognitive) tasks (e.g. Duncan, 2010; Fox et al., 2005). Here, recent studies suggest that the DAN dynamically adapts its functional connections with task-specific brain regions, or that the DAN is involved in a core function underlying multiple tasks. In this context, alternate terms have been used for the same network, such as the ‘fronto-parietal network’ or the ‘fronto-parietal control network’ (Bassett et al., 2011; Cole et al., 2013; Cona & Scarpazza, 2019; Ptak, Schnider, & Fellrath, 2017). Future research should clarify how the regions of this network interact with one another and with other brain regions to support cognitive functions such as selective attention and VSTM.

### 4.3. Conclusion

This study showed that the DAN has a key role in suppressing distracters based on features. Current findings may be important for studies aiming at disentangling different attentional processes (such as selective attention, distractibility and sustained attention) through computational modeling and at establishing their neural bases. Such modeling work could help developing a more complete account of attention and related impairments, as for instance those occurring during normal aging or after acquired brain injury.
